# Construction and validation of a preoperative malignancy risk prediction model for ovarian-adnexal masses based on clinical and ultrasonographic features

**DOI:** 10.3389/fmed.2026.1779209

**Published:** 2026-06-11

**Authors:** Liyan Zhang, Li Xiao, Huan Yang, Haiyan Wang

**Affiliations:** 1 Functional Examination Department of Obstetrics and Gynecology Center, General Hospital of Ningxia Medical University, Yinchuan, Ningxia, China; 2 The First Clinical Medical College, Ningxia Medical University, Yinchuan, Ningxia, China

**Keywords:** diagnostic efficacy, nomogram, ovarian-adnexal mass, prediction model, risk factors, sonographic characteristics

## Abstract

**Objective:**

To develop and validate a simple yet effective nomogram based on clinical and ultrasound factors for predicting the malignant risk of ovarian-adnexal masses and to evaluate its diagnostic performance and clinical utility.

**Methods:**

This retrospective study enrolled 930 patients with pathologically confirmed ovarian-adnexal masses (involving a total of 1,380 masses) from January 2020 to December 2025. Clinical data and ultrasound images of the patients were extracted from the hospital information system. Two gynecologic sonographers with more than 10 years of clinical experience, who were blinded to the pathological results, independently reviewed all ultrasound images, and all discrepancies in image interpretation were resolved through consensus discussion. To ensure the rigor of validation, all masses were randomly assigned to a training set (966 masses, numbered 1 to 966) and a validation set (414 masses) at an approximate ratio of 7:3 using a random function. In the training set, univariate analysis was first performed to screen for variables with statistical significance (*p* < 0.05). Multivariate logistic regression analyses was applied to explore the associations between each variable and the benign or malignant nature of ovarian-adnexal masses. Lasso regression analysis was conducted for the standardized processing and feature selection of the statistically significant variables. A nomogram for predicting the malignant risk of ovarian-adnexal masses was constructed based on the feature screening results of Lasso regression. To further validate the diagnostic efficacy of the model, the predictive indicators screened above were adopted to build classification models in the training set using four machine learning algorithms, namely Logistic Regression, Random Forest, XGBoost and LightGBM, and the diagnostic efficacies of these models were compared in the validation set. The area under the receiver operating characteristic curve, accuracy, sensitivity and specificity were used as the primary indicators to evaluate model performance. Internal validation of the models was completed via the bootstrap method with 1,000 resamples. Calibration curves were plotted to verify the consistency between the predicted probabilities of the nomogram and the optimal machine learning model and the actual observed results. Clinical decision curves were drawn to analyze the net benefit rate at different probability thresholds, thereby assessing the clinical practical value of the models. Finally, risk score-predicted probability calibration plots and decision plots were used for further evaluation of the prediction models to clarify their stability and clinical application value.

**Results:**

LASSO regression analysis identified 8 statistically significant independent risk factors for the malignant risk of ovarian-adnexal masses, which were categorized into four groups: clinical characteristics (menopausal status), ultrasound features (internal echogenicity, internal septations, blood flow signals), serum tumor markers (CA125, HE4), and routine blood parameters (platelet count [PLT], platelet-to-hemoglobin ratio [PHR]). A visual nomogram prediction model was successfully constructed based on these factors. Four machine learning algorithms were separately applied to the training and validation sets for comparative analysis, and the results demonstrated that all models exhibited excellent discriminatory performance: the area under the receiver operating characteristic (ROC) curve of LASSO regression analysis reached 0.97; the Logistic Regression model achieved an AUC of 0.95 in the validation set; the Random Forest model yielded AUCs of 0.95 and 0.94 in the training set and validation set, respectively; both the XGBoost and LightGBM models attained an AUC of 0.96 in both the training and validation sets. Calibration curve analysis showed a high degree of coincidence between the model-predicted curves and the ideal curve, indicating good consistency between the predicted probabilities and actual observations. Decision curve analysis revealed that the model generated significant clinical net benefits over a wide range of probability thresholds, confirming its important value in assisting clinical decision-making. Ultimately, through risk score-predicted probability calibration and decision visualization analysis, the model constructed in this study can provide a quantitative basis for formulating individualized treatment plans for patients with ovarian-adnexal masses.

**Conclusion:**

A nomogram developed to predict the malignant risk of ovarian-adnexal masses using clinical and ultrasound factors showed promising diagnostic performance. This model is framed as a hypothesis-generating tool and is intended to assist in the clinical differentiation of benign and malignant ovarian-adnexal masses, with its utility yet to be confirmed by further external validation studies.

## 1 Introduction

Ovarian cancer refers to a group of malignancies that originate in the ovaries or the related areas of the fallopian tubes and peritoneum (1). In its early stages, ovarian cancer typically lacks distinctive clinical symptoms. Some patients may only experience non-specific symptoms such as abdominal bloating, pain or gastrointestinal discomfort. Consequently, the majority of patients are diagnosed at an advanced stage—only about 15% of cases are detected early or before distant metastasis has occurred (2–4). Ovarian cancer exhibits high incidence and mortality rates globally. Statistics indicate that approximately 230,000 women are newly diagnosed with ovarian cancer worldwide each year, resulting in about 150,000 deaths. It ranks as the leading cause of death among gynecologic malignancies (5, 6). In clinical practice, the primary diagnostic approaches for evaluating ovarian-adnexal masses include ultrasonography (particularly transvaginal ultrasound) and the detection of serum tumor markers such as Carbohydrate Antigen 125 (CA125) and Human Epididymis Protein 4 (HE4). These tools play crucial roles in aiding diagnosis, assessing treatment response and monitoring for recurrence (7, 8). Ultrasound examination in the evaluation of adnexal masses is susceptible to operator experience, leading to subjective variability in diagnosing complex cases among physicians with different levels of expertise. To enhance diagnostic standardization and accuracy, the International Ovarian Tumor Analysis (IOTA) group and the American College of Radiology (ACR) have developed various risk assessment models, including the IOTA Simple Rules (SRs) and the ADNEX model (9, 10). However, these models have shown certain limitations in broader application.

Building upon the Ovarian-Adnexal Reporting and Data System (O-RADS) ultrasound lexicon established by ACR in 2018, the ACR officially released the O-RADS US Risk Stratification and Management Consensus Guidelines in 2020. This guideline provides clear clinical decision-making support through systematic terminology definitions (such as detailed descriptions of mass morphology, solid components, and blood flow signals) and a risk stratification system ranging from category 0 (incomplete assessment) to category 5 (highest risk of malignancy) (11, 12). Although the O-RADS system significantly improves the objectivity of adnexal mass assessment through its standardized lexicon and risk stratification, it remains fundamentally a morphology-based diagnostic tool, possessing inherent limitations in the specificity and precision of qualitative diagnosis (10, 13). To overcome this limitation, the current research frontier focuses on the integration of multi-dimensional information. Specifically, serum tumor markers (such as CA125 and HE4) provide biochemical signals from tumor cells (14, 15). The systemic inflammatory indicators derived from complete blood counts (such as LMR, PLR and NLR) reflect the tumor microenvironment and the host’s systemic immune response status. These indicators have been independently confirmed to correlate with the pathological progression of ovarian cancer (16, 17). Studies have shown that the absolute counts of lymphocytes and monocytes in peripheral blood and their ratio (LMR) can reflect systemic inflammation and immune status, holding significant value in predicting and assessing the prognosis of various tumors (18–20). Although these indicators are easily accessible and cost-effective, research in the field of preoperative diagnosis for ovarian malignancies remains relatively scarce, limiting their translation into routine clinical tests. To address this gap, this study, based on retrospective clinical data and utilizing methods such as logistic regression, aims to construct a preoperative risk prediction model for ovarian-adnexal malignancies. The development of this model is intended to provide substantial auxiliary support for the precise diagnosis of ovarian diseases, the personalization of treatment plans, and the improvement of clinical diagnosis and treatment efficiency through quantitative analysis.

## 2 Materials and methods

### 2.1 Patient inclusion and exclusion

**Figure 1:**
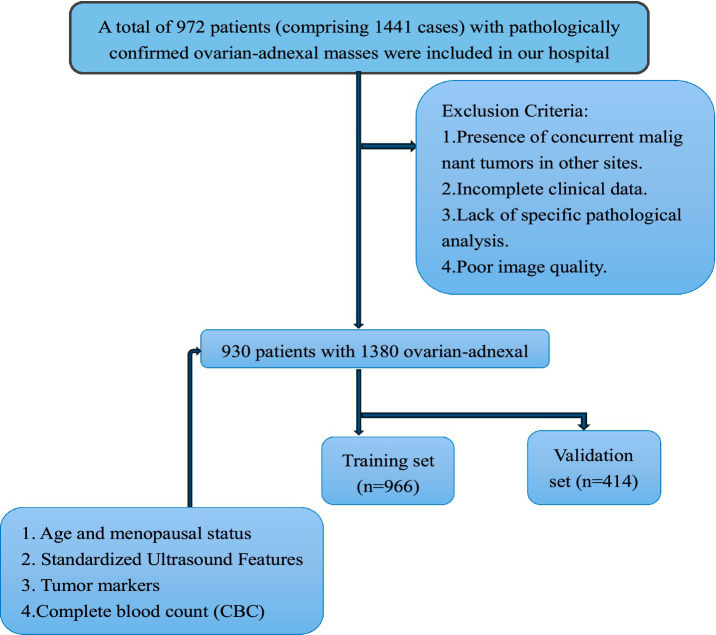
The study flowchart.

A retrospective study was conducted on 972 patients with 1,441 pathologically confirmed ovarian-adnexal mass cases at the General Hospital of Ningxia Medical University between January 2020 and December 2025. The inclusion and exclusion criteria, along with the subsequent data partitioning, are outlined below: Inclusion Criteria: If a patient had bilateral ovarian tumors, both were included. For cases with more than one tumor in a single ovary, the tumor with the larger diameter was selected if ultrasound characteristics were similar; if ultrasound features differed, the mass with more complex characteristics was chosen. Exclusion Criteria: i. Incomplete clinical data (*n* = 13) ii. Missing specific pathological classification of the lesion (*n* = 31) iii. Poor-quality images (*n* = 17). After applying these criteria, 930 patients with 1,380 masses were included in the final analysis. Data Partitioning: Lesions were randomly numbered using a random function and divided into a training set and a validation set in a 7:3 ratio. The study flowchart is shown in Figure 1. Preoperative Assessments: All patients underwent ultrasound examination within 1 week before surgery, and completed blood tests and tumor marker tests within 3 days prior to surgery. The postoperative pathological results served as the gold standard. This study was approved by the Ethics Committee of the General Hospital of Ningxia Medical University (Ethics No.: KYLL-2024-1182).

### 2.2 Data collection for included cases

#### 2.2.1 Standardized ultrasound examination

**Figure 2:**
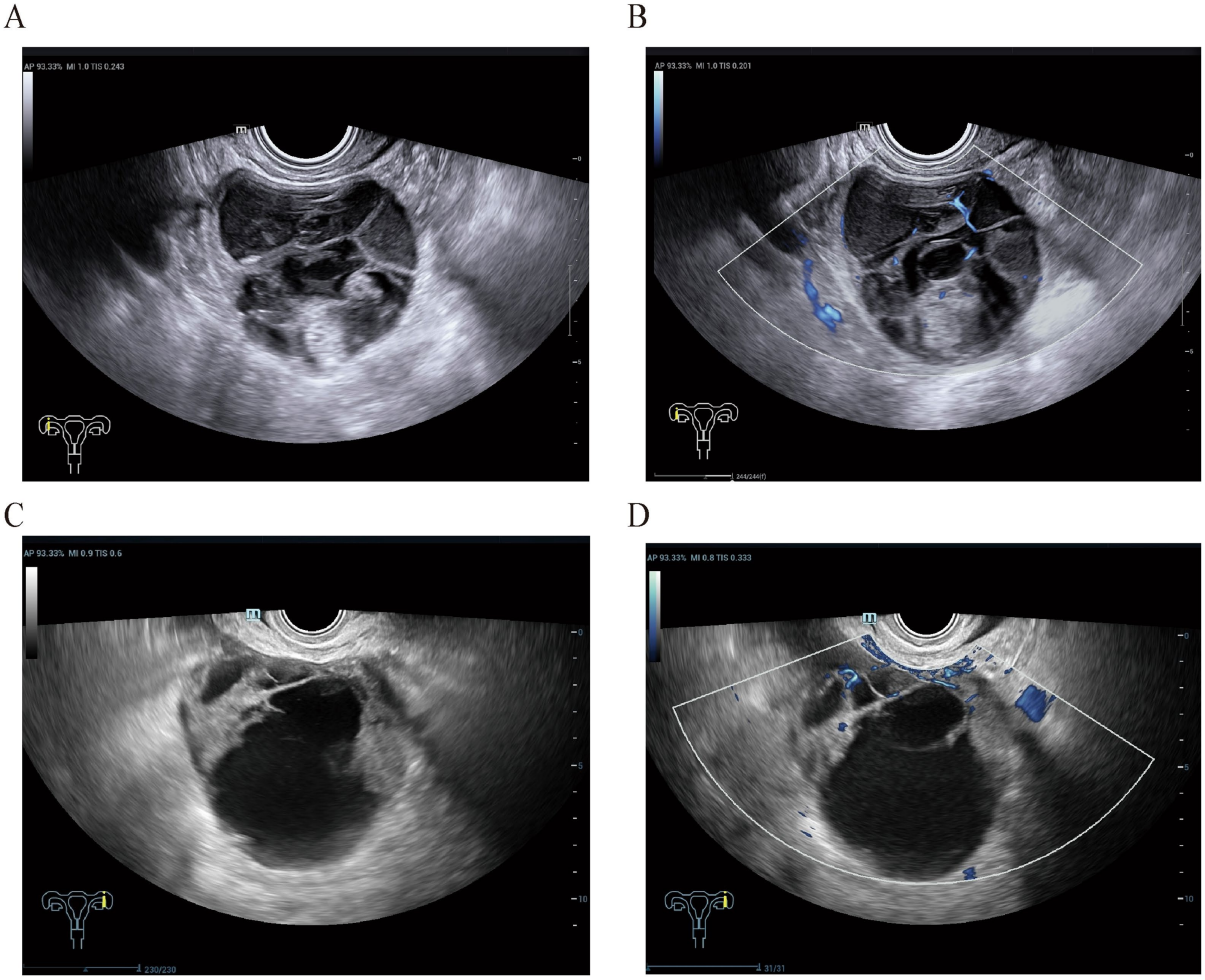
Ultrasonographic features of the ovary-adnexa. **(A)** Grayscale ultrasound findings: a cystic-solid mass, measuring approximately 8.4 × 8.1 cm, is observed superior to the left side of the uterus, with relatively clear boundaries. The cystic component demonstrates fair sound transmission. Internal septations with uneven thickness are seen. An irregular hyperechoic area protrudes from the cystic wall into the lumen, with the larger one measuring about 3.7 × 2.5 cm. **(B)** Color Doppler Flow Imaging (CDFI): a small amount of blood flow signal is detected on the septa, with a flow score of 2. Pathological diagnosis: left ovarian high-grade serous carcinoma. **(C)** Grayscale ultrasound findings: a well-defined cystic-solid mass, measuring approximately 6.5 × 5.5 cm, is seen in the right adnexal region, demonstrating poor sound transmission. The mass contains multiple locules with septations of variable thickness. A hyperechoic area is also identified within, with the larger one measuring about 1.9 × 1.2 cm. **(D)** Color Doppler Flow Imaging (CDFI): A small amount of blood flow signal is detected on the septa, with a flow score of 2. Pathological diagnosis: right ovarian multilocular mucinous cystadenoma.

Standardized ultrasound examinations were performed using a Mindray Nuewa R9 Pro ultrasound system. Patients were placed in the lithotomy position and were instructed to empty their bladders prior to the examination. A high-frequency intracavitary probe (3–10 MHz) was routinely used to conduct multiplanar and multi-azimuth scans of the uterus and bilateral adnexal regions. For cases where the lesion was particularly large or situated in a challenging location, a low-frequency abdominal probe (1–8 MHz) was additionally employed to ensure a comprehensive evaluation. All acquired ultrasound images were independently interpreted by two physicians, each with over 10 years of experience in gynecological ultrasonography. The assessments were based on the standardized definitions provided in the American College of Radiology (ACR) White Paper for the O-RADS (Ovarian-Adnexal Reporting and Data System) ultrasound lexicon. In instances of discrepant interpretations between the two physicians, a consensus opinion was reached through discussion. The following sonographic features were extracted and analyzed for this study: maximum tumor diameter (in centimeters), Lesion location (left/right/bilateral), Lesion shape (round-to-oval/irregular), Internal echogenicity (homogeneous/heterogeneous), Presence of septations (present/absent) and, if present, regularity of septations (regular/irregular), Papillary projections, defined as solid components projecting into the cystic cavity from the cyst wall or septa, with a height ≥ 3 mm, Blood flow score, assessed using a 4-point scale: 1 point for no flow; 2 points for minimal flow; 3 points for moderate flow; and 4 points for rich blood supply (21). The ultrasound image of the ovary-adnexa is shown in Figure 2.

#### 2.2.2 Data collection and laboratory procedures

Patient data, including medical history, laboratory test results, and ultrasound diagnostic information, were retrospectively collected through the electronic medical record system. The clinical data primarily included age, menopausal status, serum tumor marker levels (CA125 and HE4), and complete blood count parameters. To ensure the accuracy and clinical relevance of the test results, all laboratory procedures strictly adhered to standard operating protocols, encompassing four critical stages: patient preparation, specimen collection and processing, laboratory testing, and result verification. For the quantification of CA125 and HE4, a 5 mL sample of fasting venous blood was collected from each patient preoperatively. The sample was centrifuged at 3500 r/min for 10 min at low temperature to separate the serum. The serum levels of CA125 and HE4 were measured within 2 h of collection using a Roche cobas e602 electrochemiluminescence immunoassay analyzer, with Roche’s proprietary CA125 and HE4 detection kits employed for the analysis. The complete blood count was performed using 2–3 mL of preoperative fasting venous blood. The analysis was conducted using a five-part differential automated hematology analyzer.

### 2.3 Statistical analysis methods

All statistical analyses in this study were performed using R software (Version 4.3.3) and SPSS software (Version 27.0). Continuous variables were presented as mean ± standard deviation (xˉ ± s), and categorical variables were described as frequencies (percentages). Appropriate statistical tests were used for intergroup comparisons: the independent two-sample t-test was applied for continuous variables, and the chi-square test for categorical variables.

In the included data, univariate logistic regression analysis was first performed for each variable, and variables with a *p*-value < 0.05 were further included in the multivariate logistic regression model for secondary screening. Lasso regression was then adopted for variable feature selection, and a risk prediction model for ovarian-adnexal mass malignancy was constructed based on the key risk factors identified by Lasso regression, with the subsequent validation and performance evaluation of the model completed simultaneously. The optimal lambda value (*λ*.min) and the lambda.1se value corresponding to the simplest model were determined via 10-fold cross-validation, and the variable set corresponding to λ.min was ultimately selected for model construction.

Based on the screened variables, the SEL risk prediction nomogram was developed in this study. Meanwhile, the total samples were randomly divided into a training set and a validation set at a ratio of 7:3, and four risk prediction models (Logistic Regression, Random Forest, XGBoost, and LightGBM) were established separately, with the performance of each model evaluated in the validation set. The model evaluation metrics included the area under the receiver operating characteristic (ROC) curve (AUC), calibration curve, clinical decision curve analysis (DCA), accuracy, sensitivity, and specificity. Finally, the Local Interpretable Model-agnostic Explanations (LIME) method was integrated to conduct interpretability analysis on the optimal model, clarifying the contribution degree and effect direction of each key variable in individualized prediction.

To visualize the prediction model, a nomogram was developed using the “rms” package in R 4.3.3, with postoperative pathological results serving as the gold standard. The model’s performance was comprehensively evaluated by plotting the Receiver Operating Characteristic (ROC) curve using the “pROC” package (to assess discriminative ability), the calibration curve using the “rms” package (to assess calibration), and the Decision Curve Analysis (DCA) using the “rmda” package (to evaluate clinical utility). For all statistical tests, a *p*-value < 0.05 was considered statistically significant (significance level *α* = 0.05).

## 3 Results

### 3.1 Pathological types of 1,380 ovarian-adnexal masses

This study included a total of 1,380 pathologically confirmed ovarian-adnexal masses for analysis. Among them, 807 (58.48%) were benign masses and 573 (41.52%) were malignant masses. All cases were randomly divided into a training set and a validation set in a 7:3 ratio using a random function. The training set comprised 966 cases (565 benign, 401 malignant) and was used for model construction. The validation set contained 414 cases (242 benign, 172 malignant) and was utilized for model performance evaluation. The detailed distribution of pathological types for the 1,380 masses is shown in Table 1.

**Table 1:** Pathological types of 1,380 ovarian-adnexal masses.

Benign or malignant nature of a mass	Training set	Validation set	Total
Benign			
Mature Teratoma	121	51	172
Endometrioma	138	58	196
Serous Cystadenoma	63	28	91
Mucinous Cystadenoma	61	26	87
Simple Cyst	65	28	93
Fibroma	22	9	31
Lutein Cyst of Ovary	20	9	29
Mesosalpinx cyst	24	11	35
Hydrosalpinx/ Pyosalpinx	51	22	73
Malignant			
Borderline Tumor	41	17	58
Serous Cystadenocarcinoma	162	79	232
Mucinous Cystadenocarcinoma	138	53	197
Immature Teratoma	13	5	18
Ovarian Carcinoid	8	3	11
Clear Cell Carcinoma	13	6	19
Malignant Brenner Tumor	11	2	15
Poorly differentiated adenocarcinoma	5	3	9
Ovarian Sarcoma	4	1	5
Moderately Differentiated Ovarian Adenocarcinoma	6	3	9
Total	966	414	1,380

### 3.2 Comparison of baseline characteristics between training and validation sets

The comparative analysis of menopausal status, sonographic features, tumor markers and complete blood count parameters between the training and validation sets revealed that patients with malignant masses were significantly older than those with benign masses. Furthermore, the proportion of postmenopausal patients was significantly higher in the malignant group. These differences were statistically significant (*p* < 0.001). Regarding sonographic characteristics, benign masses typically presented with regular shapes and well-defined borders. In contrast, malignant masses were mostly irregular in shape with ill-defined borders. Internally, malignant masses were predominantly cystic-solid (mixed) or solid, often exhibiting papillary projections or cauliflower-like structures within the cystic components, accompanied by heterogeneous echogenicity with chaotic echogenic foci and dots. Benign masses, however, were primarily cystic or predominantly cystic, mostly round or oval, containing anechoic areas or fine, homogeneous echogenic dots; regular papillary echogenic foci were occasionally seen on the cyst wall. While septations could be present in benign masses, they were characteristically thin and uniform. Malignant masses displayed thick and irregular septations. Rich blood flow signals were frequently detected within and around malignant masses, whereas benign masses showed sparse flow signals, appearing as dot-like or linear signals. Additionally, the incidence of bilateral occurrence was higher in malignant masses compared to benign ones. All the aforementioned differences were statistically significant (*p* < 0.001). Analysis of laboratory indicators showed that serum levels of CA125 and HE4 were significantly elevated in the malignant group compared to the benign group (*p* < 0.001). Malignant masses can induce a systemic inflammatory state, manifested as increased neutrophils and decreased lymphocytes, leading to a higher Neutrophil-to-Lymphocyte Ratio (NLR). Platelet (PLT) counts were also higher in the malignant group. Similarly, the Monocyte-to-Lymphocyte Ratio (MLR) and Systemic Inflammatory Response Index (SIRI) were expected to be elevated due to the inflammatory state. The Platelet-to-Hemoglobin Ratio (PHR) increased accordingly, as malignant masses stimulate platelet elevation while reducing hemoglobin (HGB) levels. These differences were all statistically significant (*p* < 0.001). In the training set, the HGB level was significantly lower in malignant cases compared to benign cases (*p* < 0.05). However, in the validation set, although the HGB level in the malignant group was lower than in the benign group, the difference was not statistically significant (*p* > 0.05). The Systemic Immune-inflammation Index (SII) was higher in malignant masses compared to benign masses in both the training and validation sets, but these differences also did not reach statistical significance (*p* > 0.05). Detailed data are presented in Table 2.

**Table 2:** Comparison of baseline characteristics between training and validation sets.

Baseline characteristics	Training sets		Validation sets	
	X2	*p*	X2	*p*
average age	32.05	<0.001	10.646	<0.001
Menopausal status	19.66	<0.001	18.246	<0.001
Mass Metastasis Status	2042.196	<0.001	1498.608	<0.001
maximum inner diameter	40.888	<0.001	9.209	0.003
Mass Morphology	207.231	<0.001	80.308	<0.001
internal echo	957.723	<0.001	241.481	<0.001
Internal Septation	445.827	<0.001	195.074	<0.001
Papillary Projections>2	899.732	<0.001	560.212	<0.001
Blood flow signal	46.552	<0.001	22.836	<0.001
Location of Mass	140.487	<0.001	66.207	<0.001
CA125	207.128	<0.001	37.619	<0.001
HE4	108.394	<0.001	117.269	<0.001
NEUT%	17.87	<0.001	7.826	0.005
LYM%	10.937	<0.001	4.908	0.027
MXD%	21.158	<0.001	10.432	0.001
PLT*10-9/L	40.133	<0.001	24.526	<0.001
HGBg/L	7.972	0.005	1.268	0.261
NLR	35.937	<0.001	14.751	<0.001
MLR	68.003	<0.001	35.443	<0.001
SIRI	52.918	<0.001	44.374	<0.001
SII	0.26	0.61	0.109	0.742
PHR	39.737	<0.001	19.184	<0.001

### 3.3 Multivariable logistic regression analysis

Following the comparison of menopausal status, sonographic features, tumor markers, and complete blood count parameters between the training and validation sets (as shown in Table 2), variables with a significance level of *p* < 0.05 in the univariate analysis were further included in a multivariable logistic regression analysis. The results indicated that the following factors were independent risk factors for ovarian cancer risk: Menopausal status, Internal echogenicity of the mass, Internal septations within the mass, Presence of >2 papillary projections, Blood flow characteristics, Location of the mass (unilateral vs. bilateral), CA125 level, HE4 level, Platelet count (PLT, ×10^9^/L), Platelet-to-Hemoglobin Ratio (PHR). The detailed data are presented in Table 3. Based on the aforementioned factors, a linear prediction scoring formula was constructed as follows: Score = Internal echogenicity (score for heterogeneous: 5.29) + Internal septations (0, absent: −2.05; 1, thin and uniform: 0.81; 2, thick and irregular: 14.78) + Papillary projections (≤2: 1.40; 1, >2: 7.74) + Blood flow (Grade 1: −3.80; Grade 2: 89.22; Grade 3: 99.82; Grade 4: 100) + Mass location (0, unilateral: 1.76; 1, bilateral: 4.91) + Menopausal status (0, premenopausal: 1.77; 1, postmenopausal: 9.71) + CA125 (0, within normal range: 1.84; 1, elevated: 10.12) + HE4 (0, within normal range: 1.62; 1, elevated: 8.89) + PLT (score for values within normal range: 0.003; score for abnormal range: 0.02) + PHR (score for values within normal range: 0.11; score for abnormal range: 6.20).

**Table 3:** Multivariable logistic regression analysis.

Characteristics	Training sets		Validation sets	
	X2	*p*	X2	*p*
Menopausal status	16.629	<0.001	6.937	0.008
internal echo	8.561	0.003	5.007	0.025
Internal Septation	12.597	<0.001	10.496	0.001
Papillary Projections>2	8.355	0.004	5.284	0.022
Blood flow signal	37.68	<0.001	22.371	<0.001
Location of Mass	10.455	0.001	1.529	0.589
CA125	41.365	<0.001	10.973	<0.001
HE4	17.234	<0.001	8.385	0.004
PLT*10-9/L	6.209	<0.001	5.272	0.012
PHR	4.898	0.027	4.974	0.039

### 3.4 Lasso regression for variable screening and diagnostic performance

**Figure 3:**
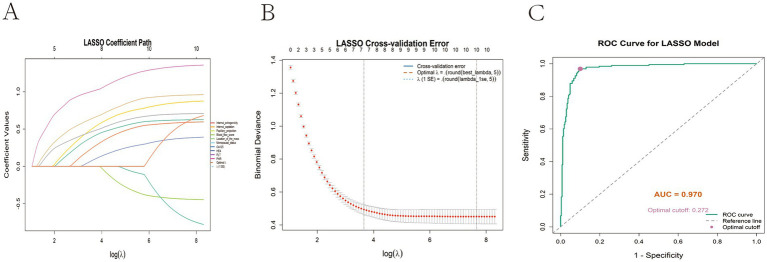
Lasso regression-based variable screening and diagnostic performance evaluation of the ovarian-adnexal mass malignancy prediction model. **(A)** LASSO coefficient path plot. **(B)** LASSO cross-validation error plot. **(C)** ROC curve for the LASSO regression model.

To identify the optimal predictive variables for the malignant risk of ovarian-adnexal masses, we performed Lasso regression analysis. The coefficient path plot (Figure 3A) demonstrated the dynamic changes in variable coefficients as the regularization parameter log(*λ*) increased, showing that some variables were gradually eliminated from the model as the penalty strength increased. The optimal regularization parameters λmin (corresponding to the lowest binomial deviance) and λ1se (corresponding to the simplest model with performance within 1 standard error of λmin) were determined via 10-fold cross-validation (Figure 3B). We selected the λmin criterion for model construction, which ultimately retained 10 statistically significant predictive variables. The diagnostic performance of the Lasso regression model was evaluated using the receiver operating characteristic (ROC) curve (Figure 3C), which yielded an area under the curve (AUC) of 0.970, confirming that the model has excellent discriminatory ability to distinguish between benign and malignant ovarian-adnexal masses. LASSO regression analysis revealed that menopausal status, ultrasound features (internal echogenicity, internal septations, blood flow signals), tumor markers (CA125, HE4), and routine blood parameters (platelet count [PLT], platelet-to-hemoglobin ratio [PHR]) were the key influencing factors for malignant ovarian-adnexal masses.

### 3.5 Develop a visual risk prediction model for malignant ovarian-adnexal masses

**Figure 4:**
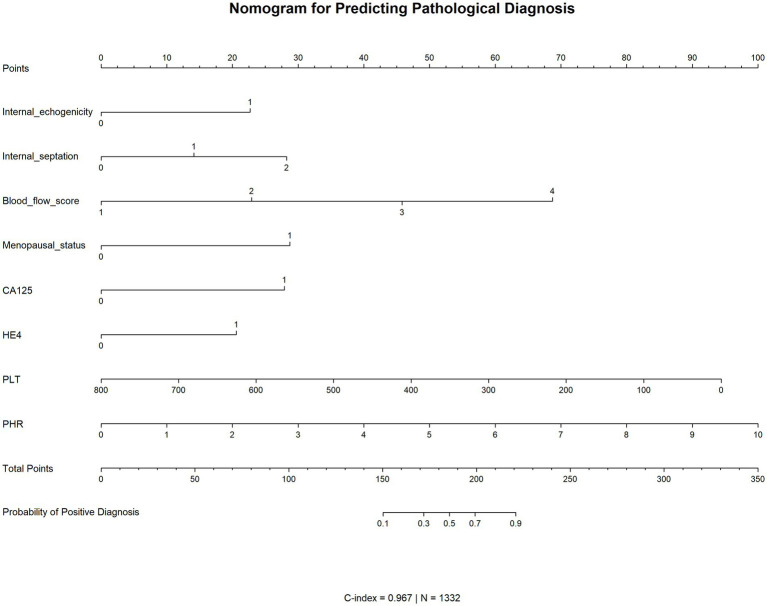
Nomogram for risk prediction based on independent risk factors.

The use of a nomogram follows a standardized scoring process designed to translate a patient’s various clinical indicators into an intuitive probability of malignancy risk. First, precisely locate the patient’s menopausal status, the sonographic features of the mass (including internal echogenicity, internal septations, and blood flow characteristics), tumor markers (CA125, HE4), and complete blood count parameters (PLT [×10^9^/L], PHR) on their corresponding variable axes of the nomogram. Then, from each variable axis, project upwards to the top “Points” scale. Read and record the individual score corresponding to each variable. Second, Sum the individual scores of all variables to obtain the Total Points. Third, Locate the Total Points on the “Total Points” axis at the bottom of the nomogram. From this point, drop a vertical line downwards. The value indicated by the intersection of this line with the bottom Risk Probability axis is the predicted probability of malignancy for the adnexal mass. The nomogram is showed in Figure 4.

### 3.6 Evaluation of the diagnostic performance of different models in the training and validation sets

All four models (Logistic Regression, Random Forest, XGBoost, LightGBM) demonstrated excellent diagnostic performance on both the training and validation sets. Specifically, the Logistic Regression model achieved an AUC of 0.95 on the validation set. The Random Forest model yielded AUC values of 0.95 on the training set and 0.94 on the validation set. Both XGBoost and LightGBM models attained AUC values of 0.96 on both the training and validation sets. These results indicate that all models possess strong discriminatory power.

Regarding model stability, the Logistic Regression model showed highly overlapping ROC curves between the training and validation sets, with AUC values consistently around 0.95, suggesting remarkable stability and no significant signs of overfitting. In contrast, the Random Forest, XGBoost, and LightGBM models exhibited perfect fit on the training set. Although their AUCs on the validation set were slightly lower than those on the training set, the values remained high, indicating potentially slight overfitting but still robust overall generalization capability. In terms of classification performance metrics, all four models achieved accuracy, sensitivity, and specificity rates above 84% in both the training and validation sets, demonstrating comprehensive and robust predictive power. To further assess the reliability of the models, internal validation was performed using the Bootstrap resampling method (with 1,000 repetitions). The calibration curves for all four models indicated a high degree of consistency between the predicted probabilities and the actual probabilities. The predicted curves showed strong agreement with the ideal curve and the calibration curve (with Hosmer-Lemeshow test *p* > 0.05), indicating good model calibration.

**Figure 5:**
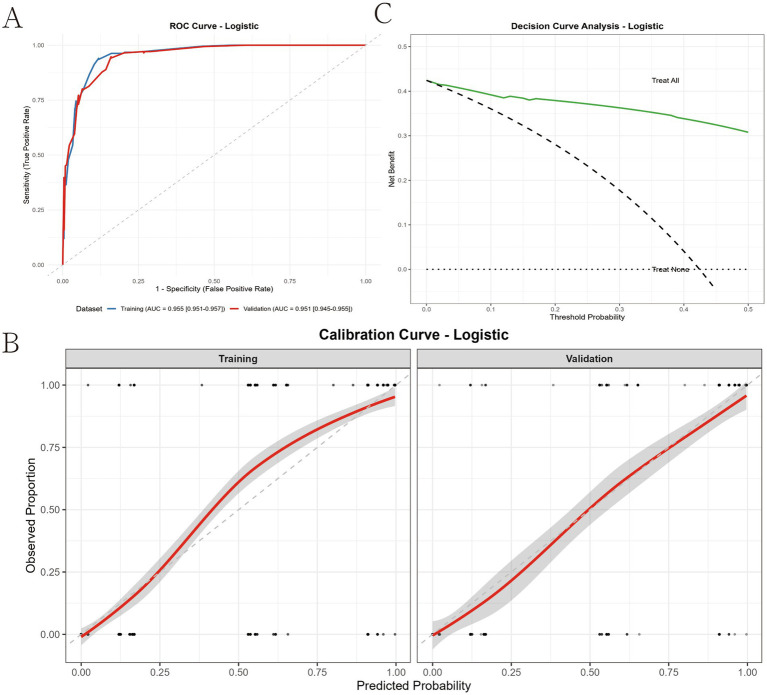
Logistic model **(A)** ROC curve, **(B)** calibration curve, **(C)** decision curve.

**Figure 6:**
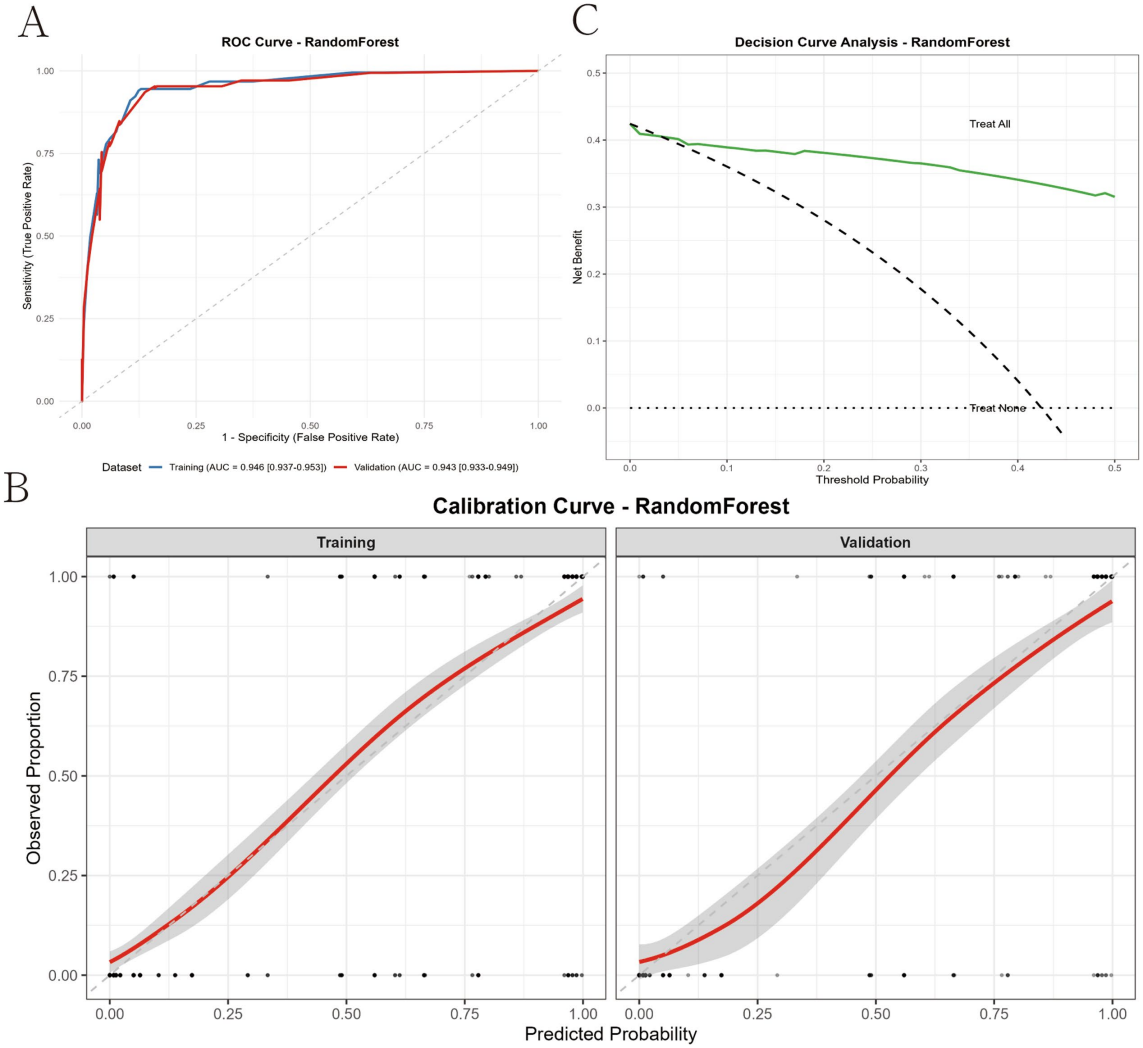
Random forest model **(A)** ROC curve, **(B)** calibration curve, **(C)** decision curve.

**Figure 7:**
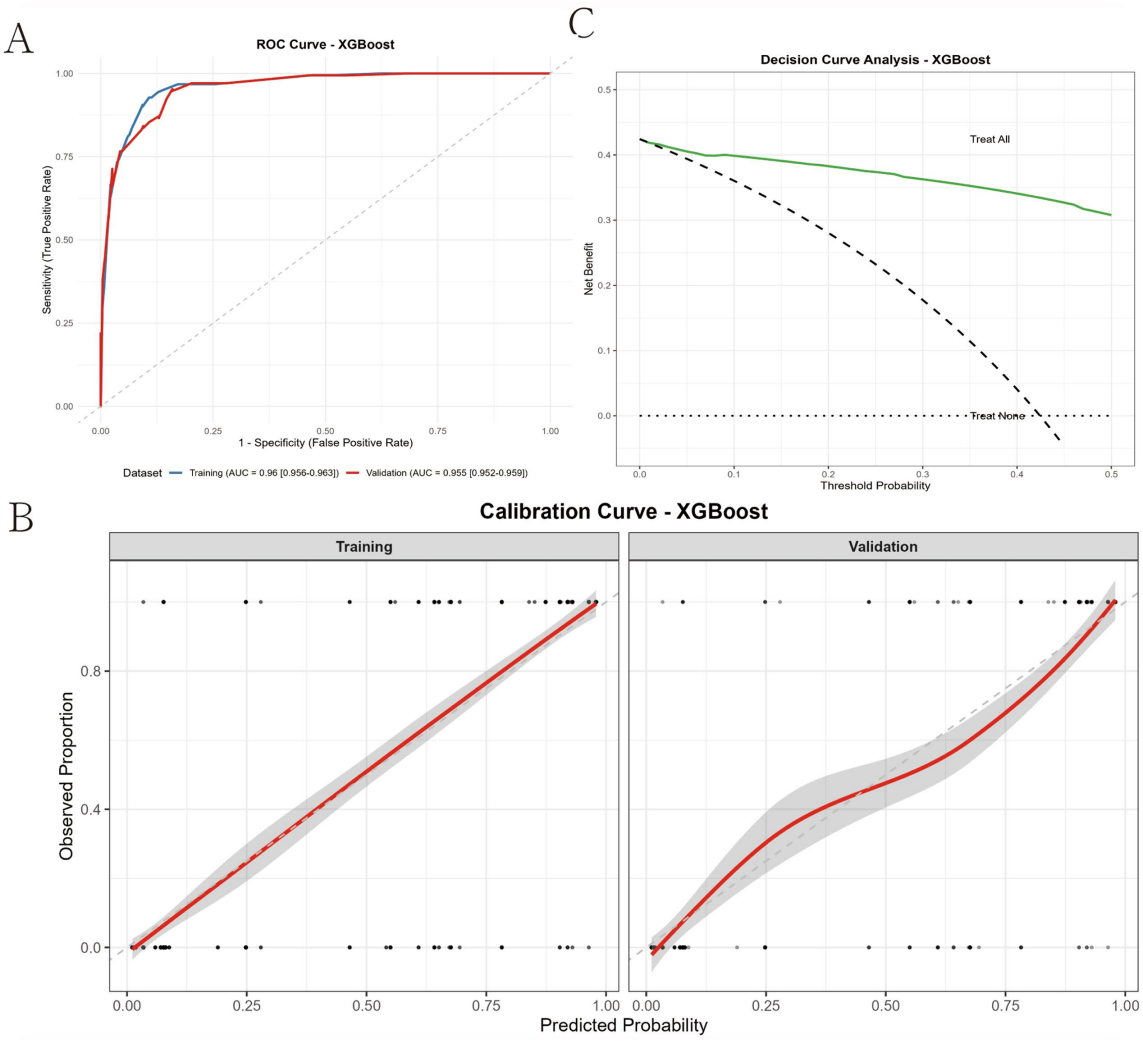
XGBoost model **(A)** ROC curve, **(B)** calibration curve, **(C)** decision curve.

**Figure 8:**
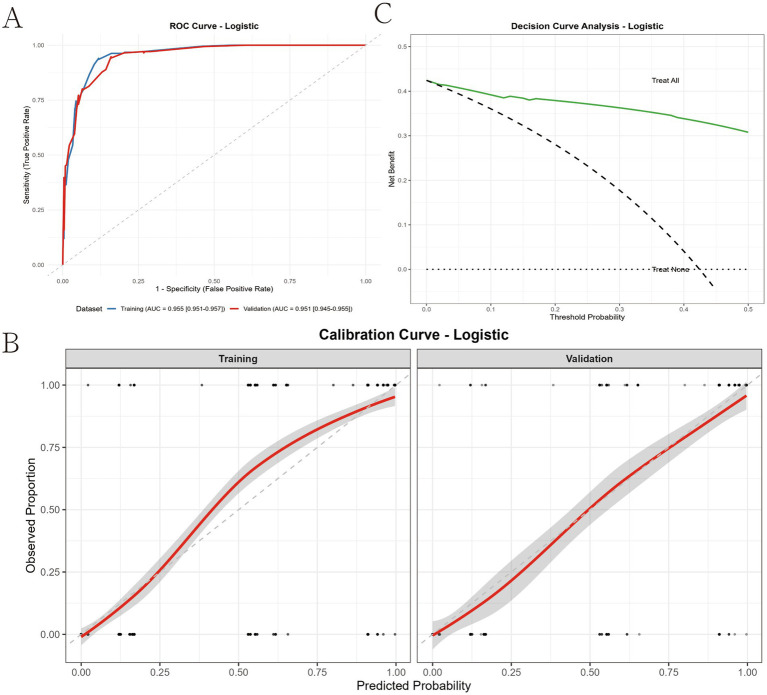
LightGBM model **(A)** ROC curve, **(B)** calibration curve, **(C)** decision curve.

The clinical practical value of the models was assessed using Decision Curve Analysis (DCA). At a threshold probability of 0.05, the decision curves of all four models were significantly higher than the reference lines of the “Intervene All” and “Intervene None” strategies. This demonstrates that employing these models for clinical decision-making can provide a clear net benefit across a relatively wide range of threshold probabilities, highlighting their significant clinical application value. The detailed data are presented in Table 4 and Figures 5–8.

**Table 4:** Comparison of diagnostic models in training and validation sets.

Model	Dataset	Accuracy	Sensitivity	Specificity	AUC	AUC_95%CI
Logistic	Training	0.904	0.935	0.881	0.96	0.955 (0.951–0.957)
Logistic	Validation	0.883	0.942	0.841	0.95	0.951 (0.945–0.955)
RandomForest	Training	0.901	0.910	0.894	0.95	0.946 (0.937–0.953)
RandomForest	Validation	0.891	0.912	0.875	0.94	0.943 (0.933–0.949)
XGBoost	Training	0.904	0.928	0.887	0.96	0.960 (0.956–0.963)
XGBoost	Validation	0.883	0.924	0.853	0.96	0.955 (0.952–0.959)
LightGBM	Training	0.906	0.905	0.907	0.96	0.962 (0.958–0.964)
LightGBM	Validation	0.871	0.871	0.871	0.96	0.957 (0.952–0.96)

### 3.7 Using the risk score-predicted probability calibration and decision graph to further evaluate the prediction model

**Figure 9:**
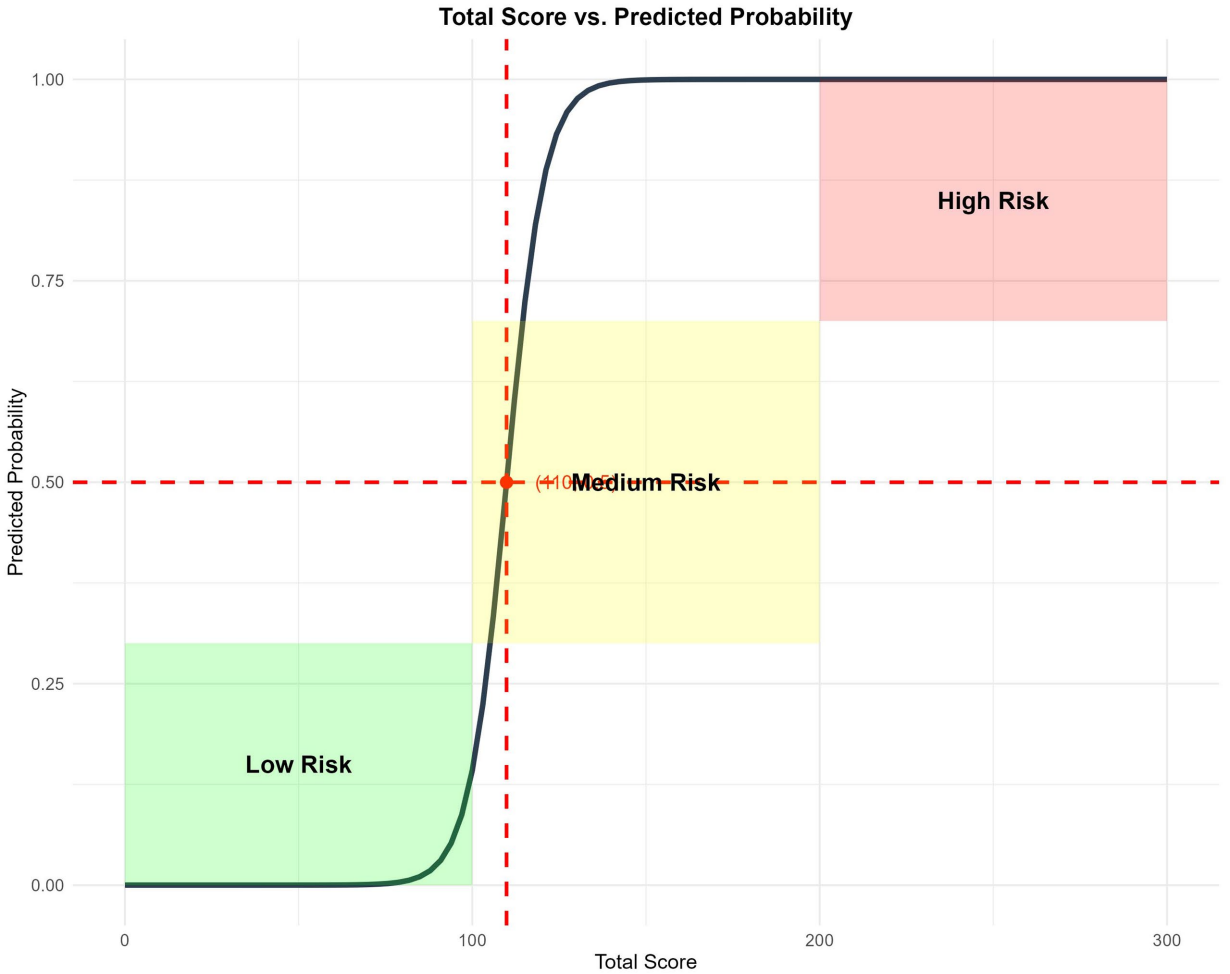
The risk score-predicted probability calibration and decision graph to evaluate the prediction model.

Different color zones in the figure represent distinct risk levels: Low Risk (Green Zone): Indicates a low total score and correspondingly low predicted probability, typically suggesting a healthy individual or a patient in a stable condition. For this group, routine health management is recommended. Unnecessary examinations and treatments should be avoided to prevent healthcare resource waste and potential iatrogenic harm. Medium Risk (Yellow Zone): Indicates a predicted probability within a moderate range (often using 0.5 as a reference threshold). Clinicians are advised to enhance monitoring and consider further differential diagnostic tests to clarify the condition. High Risk (Red Zone): Indicates a high total score and correspondingly high predicted probability, signifying a high-risk or critically ill population requiring immediate intervention. The initiation of intensive treatment measures is strongly recommended. The predicted probability is calculated from the total score using the following formula: Probability = 1 / (1 + exp.(−(−20.0069 + Total Score × 0.1821))). The detailed data are presented in Figure 9.

## 4 Discussion

In gynecologic malignancies, ovarian cancer has the highest mortality rate, making improving the accuracy of qualitative diagnosis for benign and malignant ovarian-adnexal masses a key focus of current research (22). With the continuous development of ultrasound diagnostic models, the ability to differentiate between benign and malignant ovarian-adnexal masses has seen ongoing breakthroughs. However, apart from the ADNEX model, which incorporates three clinical indicators—patient age, serum CA125 level, and type of medical center—most models still rely solely on ultrasound imaging features (23–25), resulting in certain limitations for their broad clinical application (26).

In the qualitative diagnosis of ovarian-adnexal masses, Carbohydrate Antigen 125 (CA125) and Human Epididymis Protein 4 (HE4) are commonly used clinical tumor markers (27). However, single indicators exhibit significant limitations in predicting malignant ovarian-adnexal masses. In recent years, with the continuous development of combined diagnostic strategies, the integration of ultrasound features with tumor markers has become a primary approach to enhance diagnostic specificity and sensitivity (28). For instance, Qing et al. demonstrated that the combined application of Alpha-fetoprotein (AFP), Carcinoembryonic Antigen (CEA), Carbohydrate Antigen 199 (CA199), CA125, and HE4 resulted in superior sensitivity and negative predictive value compared to any single indicator alone, suggesting that multi-marker combined Detection is more conducive to the early diagnosis of ovarian cancer (29). Additionally, studies by Winarno, Marchetti and others have further confirmed the advantage of combined detection through the integration of routine blood parameters such as neutrophil and monocyte counts (30–32).

This study is based on clinical data from 1,380 patients with ovarian-adnexal masses (807 benign cases and 573 malignant cases), integrating ultrasound features, tumor markers, and complete blood cell parameters. Through univariate and multivariate logistic regression analysis, 10 independent predictors were identified. LASSO regression analysis was further performed, and the results revealed that menopausal status, ultrasound features (internal echogenicity, internal septations, blood flow signals), tumor markers (CA125, HE4), and routine blood parameters (platelet count [PLT], platelet-to-hemoglobin ratio [PHR]) were the key influencing factors for malignant ovarian-adnexal masses.

The approach of combining ultrasound features with tumor markers for model development in this study is consistent with previous related research (8, 33–35). In this study, LASSO regression was further employed for clustering analysis to accurately identify factors contributing to clustering from a large set of candidate variables through automated variable selection and coefficient shrinkage, thereby developing a concise, stable and interpretable predictive model.

This study is the first to incorporate platelet-related indicators—Platelet Count (PLT) and the Platelet-to-Hemoglobin Ratio (PHR)—into a predictive model for ovarian cancer diagnosis. The results demonstrated that PLT levels were significantly higher in patients with malignant masses compared to those with benign masses, which aligns with the biological mechanism of platelet involvement in tumor growth, invasion, and metastasis within the tumor microenvironment (36). Evidence from the literature indicates that the incremental value of hematological parameters for ultrasound models is not absolute but exhibits significant context-dependency. Their primary utility lies in assisting the evaluation of sonographically indeterminate intermediate-risk lesions. By complementing and integrating imaging with laboratory indicators, they refine the precision of risk stratification. In clinical practice, over-reliance on combined diagnostics should be avoided. For lesions already clearly stratified as high or low risk by ultrasound alone, routine addition of hematological testing offers limited clinical benefit. Furthermore, vigilance is required against potential false positives of markers like CA125/HE4 in benign conditions such as endometriosis or pelvic inflammatory disease. Consequently, clinical decision-making is recommended to transcend reliance on any single imaging or biomarker modality. Instead, it should systematically integrate multidimensional information—including patient age, menopausal status, and medical history—to construct a more robust risk assessment framework (37–39). Concurrently, PHR was also significantly elevated in the malignant group, suggesting the presence of a tumor-related inflammatory response and potential anemic conditions. Ovarian cancer is frequently accompanied by intra-abdominal hemorrhage, coagulation dysfunction, and bone marrow hematopoietic microenvironment alterations. As a hematological indicator reflecting post-hemorrhage hematopoietic compensation/coagulation-fibrinolysis balance, changes in PHR expression levels can directly mirror the tumor burden, invasiveness, and microenvironmental abnormalities of ovarian cancer. This constitutes the biological basis for PHR to serve as a biomarker for ovarian cancer (40, 41). These findings provide new hematological biomarkers for ovarian cancer diagnosis and hold potential clinical translational value. In terms of model performance, the predictive model constructed in this study maintained high sensitivity while also improving specificity, which is likely attributable to the comprehensive inclusion of multidimensional indicators (including menopausal status, ultrasound features, and hematological parameters).

The final model showed significantly enhanced diagnostic efficacy, with the area under the receiver operating characteristic curve (AUC) exceeding 0.94 across all four models—Logistic Regression, Random Forest, XGBoost, and LightGBM—in both the training and validation sets demonstrated excellent diagnostic performance, with calibration curves showing high concordance between the predicted and ideal curves, indicating strong consistency between predicted values and actual observations. Decision curve analysis revealed that the model provided significant clinical net benefit across a wide range of probability thresholds, confirming its favorable clinical applicability. Finally, the prediction model was further evaluated through risk score–predicted probability calibration and decision plots to determine the appropriate clinical treatment strategies for masses.

### 4.1 Limitations of the study

This study has several limitations. First, the particularity of the referral model determines that the case composition of the study population tends to be high-risk groups. The patients with ovarian-adnexal masses received by our center are mainly from vertical referrals, all of whom are cases of critical, severe and complicated diseases that cannot be treated by primary hospitals and municipal hospitals in the jurisdiction. Second, the specialized diagnosis and treatment positioning of our center further strengthens the high-risk attribute of the cases. As the core institution for gynecological oncology diagnosis and treatment in the region, our center focuses on the diagnosis, treatment and research of malignant tumors such as ovarian cancer, and has a complete diagnostic system including imaging examination, pathological diagnosis and multidisciplinary collaborative diagnosis and treatment. Second, the included hematological indicators are primarily routine test items and do not involve more in-depth molecular markers. Third, the external validation of the model was conducted only at a single center, necessitating further validation in multi-center, large-sample cohorts.

### 4.2 Directions for future research

Based on the findings and limitations of this study, future research will focus on the following aspects: First, In the future, we will first verify the model efficacy through multi-center studies, cooperate with primary hospitals, municipal hospitals and other provincial Grade A tertiary hospitals in the region, include patients with ovarian-adnexal masses with different clinical settings and case compositions, expand the sample size and optimize the case structure, verify the diagnostic efficacy of this model in non-high-risk populations, and adjust the model parameters to improve its external applicability; second, we will further refine the stratified analysis of cases, divide the study population into high-risk groups and general groups according to indicators such as mass size, patient age and symptom performance, analyze the diagnostic performance of the model in different subgroups respectively, and clarify the applicable population scope of the model; finally, we will combine the specialized advantages of our center to supplement clinical diagnosis and treatment suggestions under the background of relatively high incidence of malignant tumors, provide reference for the referral of high-risk cases in primary hospitals and municipal hospitals, and provide targeted guidance for the application of the model in different clinical settings, so as to realize the hierarchical promotion and rational application of the model. Last but not least. We will translate the model into a clinically applicable decision support tool, which will be facilitated through mobile applications or online platforms to ensure its convenient clinical application.

In conclusion, this study constructed a nomogram for predicting the benign or malignant risk of ovarian-adnexal masses by analyzing a large sample of patients’ data, including age, menopausal status, ultrasound features, tumor markers, and complete blood cell counts. The study initially evaluated the predictive performance of the model on both training and validation sets using four different algorithms—Logistic Regression, Random Forest, XGBoost (Extreme Gradient Boosting), and LightGBM (Light Gradient Boosting)—and verified the preliminary diagnostic accuracy of the model using key evaluation metrics such as the area under the receiver operating characteristic curve (AUC) and calibration curves. It should be clearly stated that this model is framed as a hypothesis-generating tool. Its ability to stratify mass risk levels, demonstrated through risk score-probability calibration and decision curve analysis (DCA), only suggests its potential clinical application prospects. The actual clinical value of this model still needs to be confirmed by further external validation studies to improve its external validity and clinical applicability.

## Data Availability

The raw data supporting the conclusions of this article will be made available by the authors, without undue reservation.
